# Dietary Polyphenols and Selected Nutraceuticals in Hepatocellular Carcinoma: Mechanistic Insights, Translational Evidence, and Clinical Prospects

**DOI:** 10.3390/nu18111783

**Published:** 2026-05-31

**Authors:** Fareeha Arshad, Arshiya Akbar, Raja Chinnappan, Mohammed Imran Khan, Ahmed Yaqinuddin, Itika Arora

**Affiliations:** 1 College of Medicine, Alfaisal University, Riyadh 11533, Saudi Arabia; farshad@alfaisal.edu (F.A.); 700050001@uaeu.ac.ae (A.A.); rchinnappan@alfaisal.edu (R.C.); mikhan@kfshrc.edu.sa (M.I.K.); ayaqinuddin@alfaisal.edu (A.Y.); 2 King Faisal Specialist Hospital and Research Center, Jeddah 23433, Saudi Arabia

**Keywords:** curcumin, dietary polyphenols, gut–liver axis, hepatocellular carcinoma, liver fibrosis, MASLD, Mediterranean diet, nutraceuticals, resveratrol

## Abstract

Background: Hepatocellular carcinoma (HCC) develops predominantly from chronic liver injury, with diet representing a clinically actionable yet mechanistically complex modulator of hepatic carcinogenesis. Despite advances in immunotherapy, long-term survival remains poor, underscoring the need for complementary preventive and adjunctive strategies. Methods: We conducted a narrative review of epidemiological, experimental, and clinical literature on dietary patterns, polyphenols, and non-polyphenol nutraceuticals for HCC prevention and management, with a focus on underlying molecular and cellular mechanisms. Results: Dietary polyphenols and selected nutraceuticals exert pleiotropic effects on signaling pathways implicated in HCC, including NF-κB, STAT3, TGF-β/SMAD, PI3K/AKT, and Wnt/β-catenin, while modulating hepatic stellate cell activation, immune cell polarization, and microbiome-derived metabolites. Preclinical studies suggest that some compounds may enhance antitumor immunity and sensitize tumors to systemic therapies; however, clinical translation is constrained by limited bioavailability, pharmacokinetic variability, formulation heterogeneity, and a lack of high-quality trials. Conclusions: This review highlights the potential of dietary patterns and nutraceuticals in HCC prevention and as adjunctive therapies. It outlines key translational priorities, including etiologic stratification, biomarker-driven trial design, and rigorous safety evaluation.

## 1. Introduction

Hepatocellular carcinoma (HCC) is the predominant form of primary liver cancer, accounting for approximately 75–85% of hepatic malignancies and remaining a leading cause of cancer-related mortality worldwide, with approximately 758,000 deaths in 2022. Despite advances in surveillance, locoregional therapies, and systemic treatment, including multi-kinase inhibitors and immune checkpoint inhibitors, long-term outcomes remain poor, particularly in intermediate and advanced disease, with five-year survival rates below 20% in most regions [1,2]. This reflects late-stage presentation, underlying cirrhosis, and the biological heterogeneity of hepatocarcinogenesis.

HCC typically develops in the context of chronic liver injury characterized by persistent inflammation, fibrosis, oxidative stress, and compensatory hepatocyte proliferation. Major etiologic drivers include chronic hepatitis B and C infection, alcohol-related liver disease, aflatoxin exposure, and, increasingly, metabolic dysfunction-associated steatotic liver disease (MASLD; formerly NAFLD) and metabolic syndrome [3,4]. Over the past decade, the global epidemiology of HCC has shifted substantially: while antiviral therapies have reduced HCV-related HCC incidence, MASLD/MASH-associated HCC has emerged as the fastest-growing cause of liver cancer, particularly in high-income countries [4,5]. Consequently, diet, metabolic dysregulation, obesity, and insulin resistance now play a central role in shaping both HCC risk and disease progression. In line with the 2023 Delphi consensus, NAFLD has been renamed metabolic dysfunction-associated steatotic liver disease (MASLD) and NASH renamed metabolic dysfunction-associated steatohepatitis (MASH); we use MASLD/MASH preferentially throughout, noting legacy terms where original studies used them.

Within this context, dietary factors represent modifiable determinants of hepatocarcinogenesis. Epidemiological studies consistently associate Western-style nutritional patterns rich in saturated fat and fructose with hepatic steatosis, lipotoxicity, oxidative stress, and MASH progression (formerly NASH), thereby increasing HCC susceptibility [5,6]. In contrast, adherence to plant-forward dietary patterns, including the Mediterranean diet, higher intake of fruits, vegetables, and whole grains, and regular coffee consumption, is associated with reduced HCC incidence through combined metabolic, anti-inflammatory, and insulin-sensitizing effects [7,8]. These observations position diet as both a driver of chronic liver disease and a plausible target for HCC prevention.

Against this nutritional backdrop, dietary polyphenols and other nutraceuticals have attracted interest as potential agents for HCC chemoprevention and adjunctive therapy. Polyphenols exert pleiotropic effects relevant to hepatocarcinogenesis, including modulation of oxidative stress, inflammatory and fibrogenic signaling, lipid metabolism, and tumor cell survival [9,10]. Mechanistically, these compounds intersect key pathways implicated in HCC development and progression, such as NF-κB, STAT3, TGF-β/SMAD, PI3K/AKT, and Wnt/β-catenin, and influence hepatic stellate cell activation, immune cell polarization, and gut–liver axis signaling [11]. Preclinical studies further suggest that selected polyphenols may enhance antitumor immunity and sensitize tumors to systemic therapies; however, clinical translation is limited by poor bioavailability, pharmacokinetic variability, formulation heterogeneity, and a lack of high-quality randomized trials [12,13,14].

While prior reviews have examined polyphenol-based nutraceuticals in cardiovascular disease, general human health, or antioxidant use in HCC [15,16,17], few comprehensive syntheses have systematically evaluated both polyphenol and non-polyphenol nutraceuticals within the broader framework of chronic liver disease progression and hepatocarcinogenesis. This review therefore aims to: (i) integrate the mechanistic pathways through which diet modulates oxidative stress, immune regulation, fibrosis, lipid metabolism, and gut–liver axis signaling in hepatocarcinogenesis; (ii) evaluate dietary patterns (Mediterranean, Western, coffee) associated with HCC risk modulation; (iii) summarize representative dietary polyphenols with demonstrated relevance to HCC biology; (iv) examine selected non-polyphenol nutraceuticals (omega-3 PUFAs, vitamin E, probiotics) that modulate the metabolic–inflammatory–fibrotic continuum; and (v) outline translational priorities and clinical-trial design considerations.

Compounds were prioritized based on three criteria: (a) reproducible bioactivity across ≥2 independent preclinical HCC models, (b) availability of human pharmacokinetic or interventional data in chronic liver disease, and (c) mechanistic convergence on pathways central to hepatocarcinogenesis (NF-κB, STAT3, PI3K/AKT, TGF-β/SMAD, Wnt/β-catenin, or gut–liver axis signaling). Minerals, isolated micronutrients other than vitamin E, and non-standardized herbal mixtures were excluded.

### Literature Sources and Scope

This is a narrative review. We searched PubMed, Scopus, and Web of Science for English-language articles published between January 2000 and March 2026 using combinations of the terms ‘hepatocellular carcinoma’, ‘polyphenol’, ‘nutraceutical’, ‘Mediterranean diet’, ‘MASLD/MASH’, ‘gut–liver axis’, ‘curcumin’, ‘resveratrol’, ‘EGCG’, and ‘omega-3’. References were selected at the authors’ discretion to illustrate mechanistic, observational, and interventional evidence relevant to HCC pathogenesis, prevention, and adjunctive management. Non-English articles, conference abstracts, and case reports were not prioritized. No formal systematic screening, risk-of-bias assessment, or PROSPERO registration was performed.

## 2. Dietary Polyphenols in Hepatocellular Carcinoma

**Figure 1:**
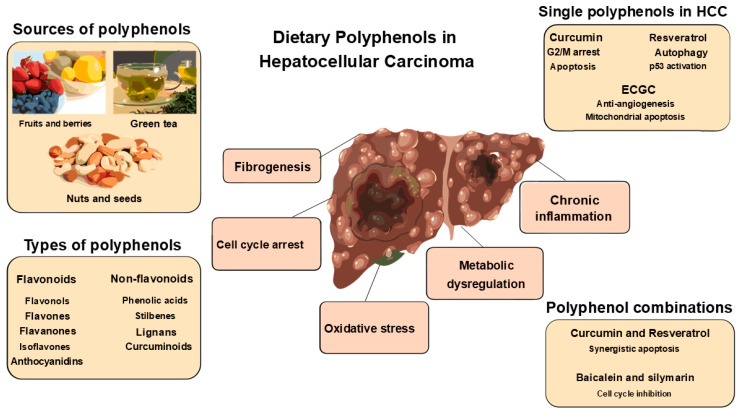
Dietary polyphenols with preventive relevance in HCC. Major flavonoids (quercetin, luteolin, baicalein, naringenin, EGCG), the curcuminoid curcumin, and non-flavonoids (resveratrol, silymarin) converge on shared hepatocarcinogenic pathways including NF-κB, STAT3, PI3K/AKT, TGF-β/SMAD, and Wnt/β-catenin signaling. Compound colors group flavonoids (blue) and non-flavonoids (green) silymarin/silibinin are flavonolignans, a structurally distinct polyphenol subclass grouped here with non-flavonoids. Abbreviations: EGCG, epigallocatechin-3-gallate; HCC, hepatocellular carcinoma; ROS, reactive oxygen species.

Dietary polyphenols are a structurally diverse class of plant-derived secondary metabolites broadly categorized into flavonoids, including flavan-3-ols, flavonols, flavones, flavanones, isoflavones, anthocyanidins, and non-flavonoids, such as phenolic acids, stilbenes, lignans, curcuminoids, and flavonolignans. These compounds are widely distributed in fruits, vegetables, whole grains, coffee, tea, cocoa, olives, nuts, herbs, and spices and make up a primary dietary source of bioactive compounds relevant to liver cancer prevention [18,19]. Functionally, polyphenols modulate oxidative stress, chronic inflammation, metabolic reprogramming, fibrogenesis, and cell-cycle regulation, key processes in the multistep progression from chronic liver injury and fibrosis to cirrhosis and hepatocellular carcinoma (HCC) [20]. As summarized in Figure 1, dietary polyphenols intersect multiple hallmarks of hepatocarcinogenesis rather than acting through a single dominant pathway. The following sections, therefore, focus on representative compounds with the strongest mechanistic and translational relevance, rather than providing an exhaustive catalog.

### 2.1. Single-Agent Polyphenols with Direct HCC Activity

#### 2.1.1. Curcumin

Curcumin, derived from *Curcuma longa*, is one of the most extensively studied polyphenols in HCC. It exerts anti-inflammatory, anti-fibrotic, antioxidant, and antitumor effects by modulating *NF-κB*, *STAT3*, *PI3K/AKT*, *MAPK*, *TGF-β/SMAD*, and *Wnt/β-catenin* signaling [21,22]. In in vitro HCC models, curcumin inhibits proliferation, induces G2/M arrest, and triggers mitochondrial apoptosis via caspase activation and regulation of the Bcl-2 family. In rodent models, it reduces tumor burden, angiogenesis, fibrosis, and oxidative stress. No phase II/III HCC clinical trials have been completed; available human data are restricted to short-duration NAFLD/MASLD trials using bioavailability-enhanced formulations and report only surrogate endpoints. Nanoformulations and targeted delivery systems markedly enhance hepatic exposure in preclinical studies, but their human pharmacokinetics, safety, and efficacy in HCC remain to be established. Preclinical evidence that curcumin sensitizes tumors to other agents suggests potential as an adjunct, but this hypothesis requires prospective clinical validation before any therapeutic recommendation [21,23].

#### 2.1.2. Resveratrol

Resveratrol (RSV) is a non-flavonoid stilbene polyphenol, structurally distinct from the flavonoid subclasses discussed above. In experimental HCC models (in vitro and rodent), resveratrol suppresses tumor initiation and progression by reducing oxidative stress, inflammation, angiogenesis, and proliferation. In vitro (HCC cell lines), it induces apoptosis and autophagy, inhibits migration and invasion, and suppresses PI3K/AKT, STAT3, and NF-κB signaling via p53 activation and PTEN upregulation [24,25,26]. Preclinical studies show that resveratrol enhances the cytotoxicity of DNA-damaging and targeted therapies while limiting oxidative injury in non-malignant tissues. No HCC-specific clinical trials of resveratrol have been reported; clinical evaluation as an adjunct remains hypothesis-generating and is further constrained by poor oral bioavailability and CYP3A4 modulation relevant to sorafenib/lenvatinib metabolism.

#### 2.1.3. Green Tea Catechins: Epigallocatechin-3-Gallate (EGCG)

In vitro, EGCG interferes with hepatocarcinogenesis by inhibiting receptor tyrosine kinases, suppressing PI3K/AKT and ERK signaling, and inducing mitochondrial apoptosis. Preclinical studies show reduced HCC cell viability, invasion, and angiogenesis. In DEN-induced rodent models, EGCG enhances sorafenib efficacy, improving tumor markers, mitochondrial function, and anti-angiogenic responses, even at low sorafenib doses. However, no completed HCC clinical trials of EGCG are available, and EFSA has flagged dose-dependent hepatotoxicity for green tea catechin extracts at ≥800 mg/day (Section 5.4); therefore, clinical positioning as a “low-toxicity adjunct” is premature.

#### 2.1.4. Flavonols and Flavones: Quercetin, Luteolin, and Baicalein

Quercetin induces apoptosis through ROS generation, mitochondrial dysfunction, caspase activation, and inhibition of NF-κB, STAT3, and PI3K/AKT signaling [27], while suppressing tumor growth and modulating the immune microenvironment in vivo [28]. Luteolin inhibits HCC growth via p53 and Fas/FasL activation and interference with TGF-β, PI3K/AKT, NF-κB, JAK/STAT, and Wnt/β-catenin pathways [29,30]. Baicalein induces apoptosis and autophagy through ER stress, caspase activation, and regulation of LC3 and Beclin-1 [31]. Together, these compounds expand the mechanistic landscape and support the rational design of polyphenol combinations [32,33].

#### 2.1.5. Citrus Flavanones: Naringenin

In vitro, naringenin induces ROS-dependent mitochondrial apoptosis, cell-cycle arrest, and inhibition of JAK2/STAT3 signaling in HCC cells. Nanoparticle formulations enhance uptake and cytotoxicity while maintaining favorable toxicity profiles in preclinical models. Human HCC data are not available, and clinical translation remains speculative [34].

#### 2.1.6. Milk Thistle Flavonolignans: Silymarin and Silibinin

Silymarin and its major component, silibinin, exhibit antiproliferative, pro-apoptotic, immunomodulatory, and hepatoprotective effects in preclinical HCC models [35,36]. In vitro (HepG2 cells), silymarin suppresses proliferation by modulating cyclins, CDKs, and the p53/p21/p27 axis. The only relevant high-quality human RCT was conducted in biopsy-proven NASH (not HCC) and failed to meet its primary histologic endpoint, although fibrosis-related signals were observed (Section 5.2). No HCC-specific clinical trials are available; any role as an adjunct remains hypothesis-generating [37].

### 2.2. Polyphenol-Rich Extracts and Diet-like Exposures

Polyphenol-rich plant extracts better reflect dietary exposure than single compounds. Grape, mulberry, *Solanum nigrum*, and olive oil extracts reduce tumor burden, oxidative stress, and liver injury in experimental models of hepatocarcinogenesis [38,39,40]. Multi-component extracts may enhance stress-response activation and selectively inhibit HCC cell migration and invasion. However, the lack of standardization and constituent attribution remains a major translational challenge [39,40].

### 2.3. Polyphenol Combinations and Polyphenol–Drug Interactions

#### 2.3.1. Polyphenol–Polyphenol Combinations

Rational polyphenol combinations exploit complementary mechanisms. Curcumin and resveratrol show synergistic antiproliferative and pro-apoptotic effects via enhanced caspase activation and ROS generation [23]. Baicalein and silymarin similarly produce additive to synergistic growth inhibition with minimal toxicity to normal hepatocytes [31,41].

#### 2.3.2. Polyphenol–Drug Combinations

Polyphenols enhance the efficacy of conventional therapies in preclinical HCC models. Quercetin potentiates cisplatin-induced apoptosis [42], while resveratrol and EGCG synergize with sorafenib to improve tumor markers, apoptosis, and anti-angiogenic effects [43,44,45]. Nano-formulated multi-polyphenol combinations further increase cytotoxicity [46,47]. However, translation is limited by supraphysiological dosing, metabolic complexity, and uncharacterized drug–nutrient interactions. Systematic pharmacokinetic and pharmacodynamic evaluation is essential before clinical integration, particularly for interactions involving cytochrome P450 enzymes (e.g., CYP3A4 modulation by resveratrol affecting sorafenib/lenvatinib metabolism), efflux transporters (P-gp, BCRP), and shared oncogenic signaling pathways [18,19,20,35].

The breadth of caspase-3, -8, -9, Bcl-2, and Survivin targeting across these compounds supports rational combinations that engage extrinsic and intrinsic pathways simultaneously, but all evidence remains preclinical.

### 2.4. Polyphenol-Mediated Cell Death Pathways in HCC

#### 2.4.1. Regulation of Apoptotic Pathways

**Figure 2:**
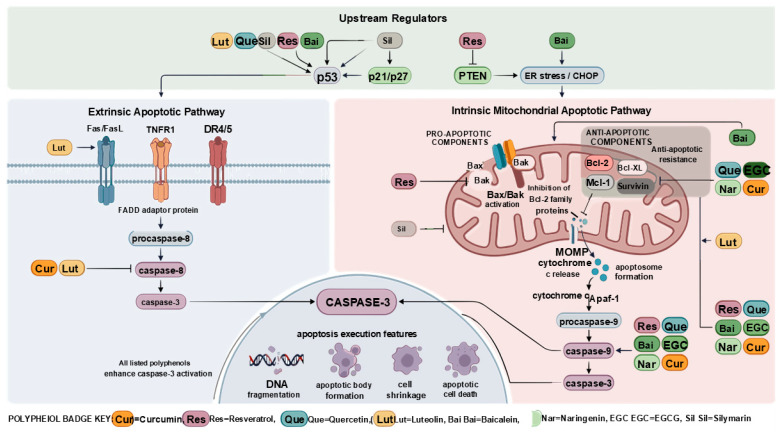
Polyphenol-mediated activation of apoptotic pathways in HCC. The extrinsic pathway proceeds through death receptors (Fas/FasL, TNFR1, DR4/5) and FADD to activate caspase-8. The intrinsic pathway proceeds through Bax/Bak-mediated MOMP, cytochrome c release, Apaf-1 assembly, and caspase-9 activation. Both converge on caspase-3, which executes DNA fragmentation, apoptotic body formation, and cell shrinkage. Bcl-2, Bcl-xL, Mcl-1, and Survivin are anti-apoptotic resistance nodes targeted by the polyphenols shown. Upstream regulators p53, p21/p27, and PTEN/AKT modulate pathway sensitivity. Abbreviations: Apaf-1, apoptosis protease-activating factor 1; DR4/5, death receptors 4 and 5; FADD, Fas-associated death domain; MOMP, mitochondrial outer membrane permeabilization; TNFR1, tumour necrosis factor receptor 1. Badge key: Cur, Curcumin; Res, Resveratrol; Que, Quercetin; Lut, Luteolin; Bai, Baicalein; Nar, Naringenin; EGC, EGCG; Sil, Silymarin. Polyphenol badges are colour-coded as follows: orange, Cur (curcumin); red, Res (resveratrol); green, Que (quercetin); yellow, Lut (luteolin); teal, Bai (baicalein); light blue, Nar (naringenin); dark green, EGQ/EGC (EGCG); grey, Sil (silymarin/silibinin). The left panel depicts the extrinsic apoptotic pathway and the right panel the intrinsic mitochondrial apoptotic pathway.

Induction of apoptosis is a key mechanism through which dietary polyphenols suppress HCC progression (Figure 2). Polyphenols regulate both the extrinsic and intrinsic apoptotic routes through modulation of Fas/FasL, TNFR1, DR4/5, caspase-8, Bax/Bak, cytochrome c release, caspase-9, and caspase-3 signaling [48]. Curcumin induces caspase-dependent apoptosis and downregulates Bcl-2-associated survival pathways in HCC models [21]. Resveratrol promotes mitochondrial apoptosis through p53 activation and inhibition of PI3K/AKT and STAT3 signaling [25]. Quercetin, luteolin, baicalein, EGCG, and naringenin enhance apoptotic sensitivity through caspase activation, ROS-mediated mitochondrial dysfunction, and suppression of anti-apoptotic mediators including Bcl-2 family proteins and Survivin [49].

#### 2.4.2. Regulation of Ferroptosis Pathways

**Figure 3:**
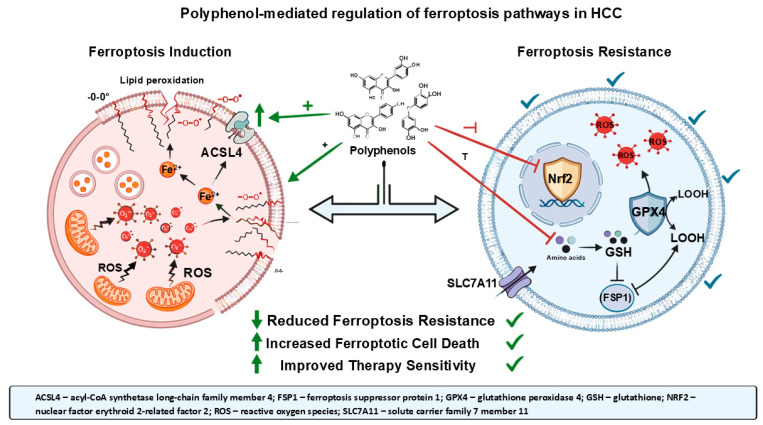
Polyphenol-mediated regulation of ferroptosis pathways in HCC. Ferroptosis induction (**left panel**) is driven by ROS accumulation, Fe^2+^-dependent lipid damage, ACSL4 upregulation, and lipid peroxidation. Ferroptosis resistance (**right panel**) is mediated by Nrf2 signaling, GSH synthesis, GPX4, SLC7A11, and FSP1. Net outcome: reduced ferroptosis resistance, increased ferroptotic cell death, and improved therapy sensitivity. Abbreviations: ACSL4, acyl-CoA synthetase long-chain family member 4; FSP1, ferroptosis suppressor protein 1; GPX4, glutathione peroxidase 4; GSH, glutathione; Nrf2, nuclear factor erythroid 2-related factor 2; ROS, reactive oxygen species; SLC7A11, solute carrier family 7 member 11. Green arrows indicate activation and enhancement, whereas red blunt-ended lines indicate inhibition or suppression. Green check marks denote favorable therapeutic outcomes associated with polyphenol-mediated modulation of ferroptosis.

Recent evidence indicates that dietary polyphenols also regulate ferroptosis, an iron-dependent form of cell death characterized by excessive lipid peroxidation and oxidative membrane damage (Figure 3). Polyphenols including curcumin, quercetin, baicalein, and EGCG have been associated with increased ROS accumulation, ACSL4 activation, Fe^2+^-dependent lipid peroxidation, and enhanced ferroptotic susceptibility in liver cancer models [50,51]. In contrast, activation of the Nrf2/GPX4/SLC7A11 antioxidant axis promotes ferroptosis resistance in HCC through enhanced glutathione synthesis and lipid peroxide detoxification [52]. Curcumin specifically promotes ferroptosis in HCC through ACSL4 upregulation and suppression of GPX4-dependent antioxidant defenses, confirmed in vitro and in a xenograft model in vivo [50]. Resveratrol, naringenin, EGCG, and silymarin have additionally been associated with modulation of Nrf2-related antioxidant signaling that influences ferroptosis sensitivity in HCC and experimental liver disease models [26,53]. Together, these findings indicate that modulation of apoptosis and ferroptosis contributes to the antitumor activity of dietary polyphenols in HCC.

## 3. Non-Polyphenol Nutraceuticals

Beyond polyphenols, several non-polyphenol nutraceuticals have been investigated for their capacity to modulate the metabolic–inflammatory–fibrotic continuum that underlies the development of most hepatocellular carcinoma (HCC), particularly in the setting of metabolic dysfunction-associated steatotic liver disease (MASLD), metabolic dysfunction-associated steatohepatitis (MASH; formerly NASH), and cirrhosis. Unlike direct anticancer paradigms, these interventions are best conceptualized as upstream disease modifiers that reduce steatosis, oxidative injury, endotoxemia, and immune dysregulation, processes that shape both HCC risk and hepatic reserve, two major determinants of long-term outcomes in chronic liver disease [54]. The principal mechanistic targets, most consistent human evidence, potential HCC-specific applications, and key safety considerations for major non-polyphenol nutraceuticals are summarized in Table 1. Collectively, these agents influence host biology rather than exerting direct cytotoxic effects on tumor cells.

**Figure 4:**
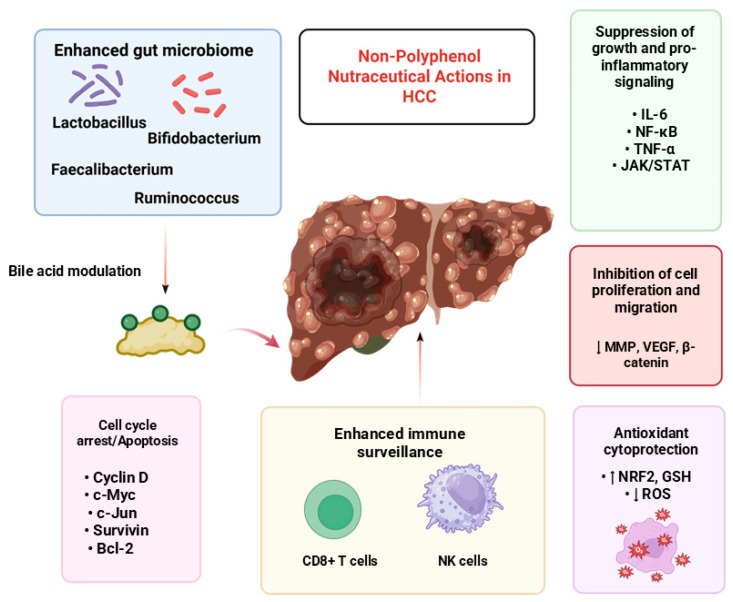
Conceptual schematic of non-polyphenol nutraceutical actions in HCC. Omega-3 PUFAs (EPA/DHA), vitamin E (α-tocopherol), and probiotics act as upstream disease modifiers along the metabolic–inflammatory–fibrotic continuum that drives MASLD/MASH-associated HCC. Arrows indicate mechanistic effects on hepatic lipid metabolism, oxidative stress, immune signaling, and interactions within the gut–liver axis. Upward arrows (↑) indicate increased activity, expression, or levels, whereas downward arrows (↓) indicate decreased activity, expression, or levels. Abbreviations: DHA, docosahexaenoic acid; EPA, eicosapentaenoic acid; HCC, hepatocellular carcinoma; MASLD, metabolic dysfunction-associated steatotic liver disease.

The upstream biological pathways through which non-polyphenol nutraceuticals modulate hepatocarcinogenesis, including lipid metabolism, oxidative stress responses, inflammatory signaling, immune surveillance, and gut–liver axis interactions, are conceptually illustrated in Figure 4.

### 3.1. Omega-3 Fatty Acids and MASLD/NASH Progression

Omega-3 polyunsaturated fatty acids (n-3 PUFAs), particularly EPA and DHA, modulate key drivers of hepatocarcinogenesis, including inflammation, lipotoxicity, oxidative stress, and immune signaling. Increased hepatic n-3 PUFA availability shifts lipid mediator production toward pro-resolving pathways, limiting chronic inflammation, fibrosis, and oncogenesis [55,56]. A UK Biobank prospective cohort study by Jiang et al. (2021) reported that habitual use of fish oil supplements was observationally associated with a reduced risk of primary liver cancer, including HCC [58]; residual confounding from healthy-user behavior cannot be excluded. This finding is complemented by Liu et al. (2024), who linked higher circulating omega-3 fatty acid biomarkers to lower HCC incidence in the same cohort [59], consistent with known biological effects. Preclinical studies further show that EPA and DHA inhibit oncogenic pathways, including cyclooxygenase-2 (COX-2) and Wnt/β-catenin signaling [72]. Clinically, omega-3s are best positioned as supportive or preventive strategies, with meta-analyses demonstrating improved postoperative outcomes following hepatectomy, including in hepatitis B-associated HCC [60,61].

### 3.2. Vitamin E and Antioxidant Modulation

Oxidative stress promotes hepatocarcinogenesis by driving lipid peroxidation, mitochondrial dysfunction, and ROS-mediated DNA damage, leading to The PIVENS trial demonstrated that vitamin E (800 IU/day) improves histologic features in nondiabetic, non-cirrhotic NASH, supporting its role as a disease-modifying therapy in selected patients [63]; PIVENS excluded patients with diabetes and cirrhosis, limiting extrapolation to HCC-risk populations. Observational studies in advanced NASH further associate vitamin E use with improved transplant-free survival and reduced hepatic decompensation [64]. However, high-dose vitamin E is linked to increased hemorrhagic stroke risk despite modest reductions in ischemic stroke [66], limiting its applicability in cirrhosis and HCC. Thus, vitamin E should be viewed as a selective NASH-targeted intervention. Note that the SELECT prostate cancer signal involved a different dose (400 IU/day) than PIVENS (800 IU/day); risk estimates are therefore not directly transferable across dosing regimens.

### 3.3. Probiotics and the Gut–Liver Axis

The gut–liver axis is mechanistically central to MASLD/MASH-driven HCC: increased intestinal permeability enables portal delivery of lipopolysaccharide (LPS) and other PAMPs that activate hepatic TLR4–NF-κB signaling, sustain Kupffer-cell and stellate-cell activation, and drive the inflammation–fibrosis–HCC sequence. Dysbiosis-derived secondary bile acids (e.g., deoxycholic acid) further amplify hepatocyte DNA damage and senescence-associated secretory phenotypes that promote hepatocarcinogenesis. In cirrhosis irrespective of etiology, microbial translocation and altered bile acid signaling have been linked both to HCC incidence and to attenuated response to immune checkpoint inhibitors. Within this HCC-specific framework, probiotic evidence remains limited but directionally consistent: a retrospective cohort study in HBV-related cirrhosis reported a dose-responsive association between probiotic exposure and reduced HCC incidence, and a Lactobacillus rhamnosus murine model demonstrated attenuation of gut–liver inflammatory pathways with reduced tumor burden. RCT-level evidence in cirrhosis supports improvements in hepatic encephalopathy and dysbiosis, with a favorable safety profile. These findings are hypothesis-generating; prospective HCC-endpoint trials with strain-defined products and microbiome/bile-acid biomarkers are required before clinical adoption [69].

## 4. Mechanistic Pathways

**Figure 5:**
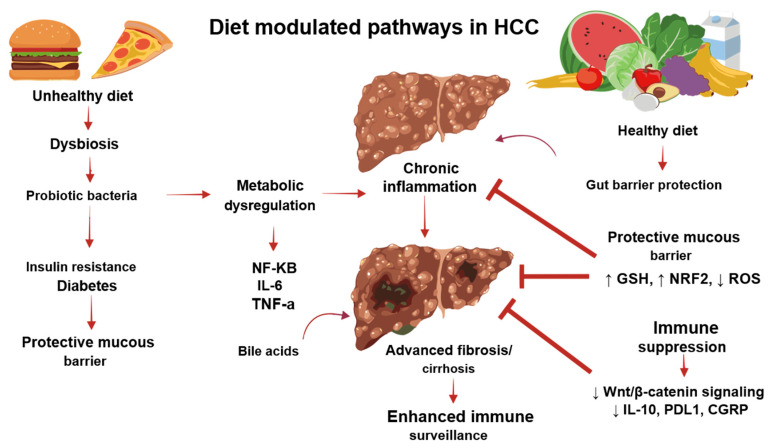
Proposed conceptual model linking dietary patterns to HCC risk. Western dietary patterns (high in saturated fat, fructose, and ultra-processed foods) drive steatosis, lipotoxicity, and oxidative stress, thereby fueling fibrogenic and oncogenic signaling. Plant-forward patterns (Mediterranean, polyphenol-rich, coffee) attenuate these axes via Nrf2 activation, anti-inflammatory lipid mediators, and microbiome diversification.Upward arrows (↑) indicate increased expression, activation, or levels, whereas downward arrows (↓) indicate decreased expression, activation, or levels. Red arrows and inhibitory bars indicate disease-promoting pathways or suppressive interactions, whereas black arrows indicate pathway progression or biological associations.. Abbreviations: HCC, hepatocellular carcinoma; ROS, reactive oxygen species.

Hepatocellular carcinoma (HCC) arises from the convergence of chronic liver injury, metabolic dysregulation, and persistent inflammatory signaling, with diet influencing each of these axes through effects on redox balance, immune tone, gut-derived signals, and lipid metabolism [73,74]. Although diet is not a single mechanistic variable, relevant dietary exposures repeatedly converge on a limited set of interconnected biological circuits: oxidative stress, cytokine-driven oncogenic inflammation, intestinal permeability and microbiome-derived metabolites, and de novo lipogenesis, which collectively create a permissive microenvironment for hepatocarcinogenesis [73,74]. A conceptual framework linking dietary patterns to these interacting pathways across the continuum from chronic liver disease to HCC is illustrated in Figure 5.

These circuits are not independent: oxidative stress amplifies inflammatory signaling and fibrogenesis; inflammatory pathways reprogram hepatocyte and immune-cell metabolism; microbiome-derived metabolites shape bile acid pools and immune tone; and lipid metabolic remodeling feeds back into redox imbalance and immune suppression [73,74]. Within this system’s context, dietary polyphenols and nutraceuticals are mechanistically attractive because they can modulate multiple nodes simultaneously [75]. However, such pleiotropy introduces translational complexity, as pathway effects are highly context- and stage-dependent [75].

### 4.1. Oxidative Stress, Redox Plasticity, and Cell Death Programs

Oxidative stress is a central driver of fibrosis, genomic instability, and hepatocarcinogenesis [76]. Hepatic ROS originate from metabolic and inflammatory sources and exert dose-dependent effects, with moderate levels promoting tumor survival and excessive levels inducing cell death [73,74]. Antioxidant capacity, regulated by the Keap1–Nrf2 pathway, determines this balance [75]. While transient Nrf2 activation is antifibrotic, sustained activation in HCC enhances redox buffering, metabolic flexibility, and therapy resistance [75]. Diet-derived antioxidants may therefore be protective in premalignant disease but potentially tumor-supportive in established HCC, underscoring the need for stage-specific approaches [73,75]. Redox control also regulates ferroptosis, an iron-dependent form of cell death in HCC. Activation of the p62–Keap1–Nrf2 axis suppresses ferroptosis by enhancing glutathione and lipid peroxide detoxification, suggesting that antioxidant-rich diets may reduce ferroptotic pressure, whereas pro-oxidant strategies could enhance treatment efficacy [52,75].

However, the feasibility and safety of pro-oxidant strategies in patients with cirrhosis, coagulopathy, or portal hypertension remain unproven and should not be pursued outside carefully designed clinical trials.

### 4.2. Inflammatory Signaling and Immune Modulation

Oxidative stress and inflammation form a self-reinforcing loop that sustains fibrosis and HCC risk [74,76]. HCC arises in a chronically inflamed, immunosuppressive microenvironment driven by IL-6/JAK/STAT3 and NF-κB signaling, which promote tumor growth, immune evasion, and myeloid polarization [74,77,78,79]. Nutritional compounds modulate inflammatory and immune circuits in HCC: curcumin and resveratrol attenuate NF-κB and STAT3 activation, EGCG and quercetin dampen IL-6/JAK signaling, and omega-3 PUFAs shift lipid mediator production toward pro-resolving pathways, although clinical translation remains limited [73,79]. Disease etiology further shapes immune responsiveness: in MASH (formerly NASH), lipotoxic stress induces T-cell exhaustion and resistance to PD-1 blockade [80]. Myeloid-driven IL-8/CXCR2 signaling contributes to immune resistance, and CXCR2 inhibition restores checkpoint sensitivity in NASH-HCC models [81]. These findings support evaluation of dietary and nutraceutical strategies as adjunctive immune modulators in selected populations [82].

### 4.3. Gut–Liver Axis, Microbiome, and Bile Acid Signaling

The gut–liver axis is integral to hepatocarcinogenesis, as microbial products delivered via the portal vein sustain hepatic inflammation and fibrogenesis in chronic liver disease [83]. Dysbiosis and impaired barrier function amplify this process, increasing HCC risk [83]. Bile acids act as signaling molecules that link microbiome composition to hepatic metabolic and immune pathways via FXR and TGR5, forming a bidirectional regulatory loop [83]. Disrupted bile acid signaling promotes inflammation, metabolic dysfunction, and immune dysregulation, fostering a carcinogenic microenvironment [83]. Gut microbiome composition is also associated with immunotherapy response in HCC, with responders to anti–PD-1 therapy exhibiting distinct microbial profiles and lower intestinal inflammation [84,85]. Multi-omics analyses further support the use of microbiome-derived biomarkers as predictors of treatment response and prognosis [86]. Given the strong influence of diet on microbiome structure, nutritional strategies targeting the gut–liver axis are particularly attractive in MASLD/MASH-HCC, where lipotoxicity-driven CD8^+^ T-cell exhaustion attenuates ICI efficacy and myeloid IL-8/CXCR2 signaling sustains immune resistance. Diet- and probiotic-mediated remodeling of microbiome composition, bile acid pools, and barrier integrity, therefore, represents a mechanistically aligned adjunctive lever specifically for the etiologic subset of HCC most resistant to current systemic therapy [80,81,83,84,85,86].

### 4.4. Lipid Metabolism, De Novo Lipogenesis, and Carcinogenesis

**Figure 6:**
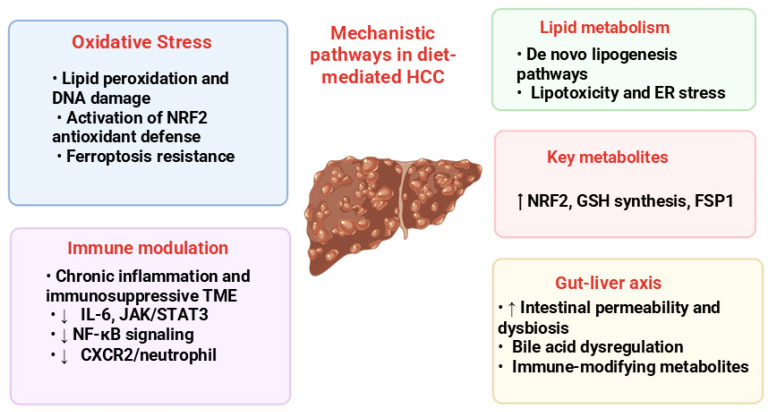
Integrated model of diet-modulated pathways in HCC. Diet and metabolites influence recurring circuits of oxidative stress, inflammation, immune signaling, disruption of the gut–liver axis, and lipid metabolic reprogramming in HCC.Upward arrows (↑) indicate increased activity, expression, or levels, whereas downward arrows (↓) indicate decreased activity, expression, or levels.

Dysregulated lipid metabolism is a hallmark of HCC and reflects both metabolic liver disease and oncogenic reprogramming [87,88]. Excess caloric intake, particularly fructose and saturated fat, drives steatosis, lipotoxicity, oxidative stress, inflammation, and fibrosis [87]. In HCC, de novo lipogenesis (DNL), regulated by SREBP-1 and enzymes including ACLY, ACC, FASN, and SCD1 (stearoyl-CoA desaturase 1), is essential for tumor growth and correlates with poor outcomes [87]. Pharmacologic inhibition of lipogenesis reduces hepatic DNL and steatosis in humans, supporting feasibility for metabolic targeting [89]. Dietary fructose promotes HCC progression via microbiota-derived acetate and increased protein O-GlcNAcylation [90]. Lipid accumulation also enhances ER stress, lipid peroxidation, ferroptosis sensitivity, and immunosuppressive inflammation [52,73,87]. Thus, reducing hepatic lipotoxicity may concurrently improve redox balance, inflammation, immune tone, and fibrosis. A synthesis of diet-responsive mechanistic axes and their translational implications is provided in Table 2, and an integrated overview of diet-modulated pathways in HCC is shown in Figure 6.

## 5. Clinical Evidence and Translational Potential

**Figure 7:**
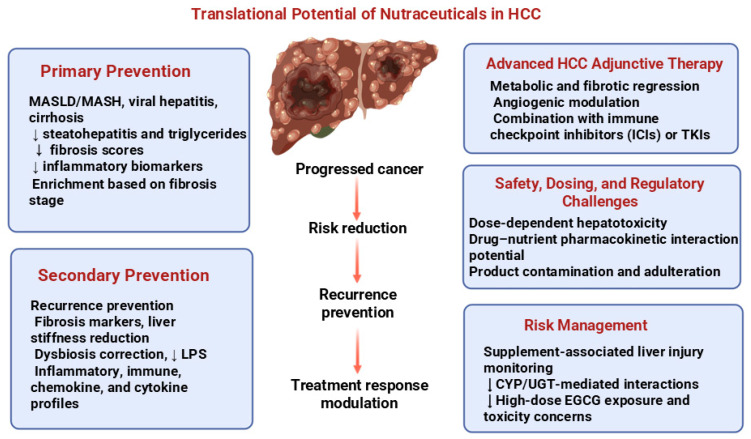
Translational roadmap for dietary nutraceuticals in HCC. The framework spans three intervention windows: (i) primary prevention in MASLD/MASH, viral hepatitis, and cirrhosis; (ii) secondary prevention following curative resection or ablation; and (iii) adjunctive use in advanced disease alongside systemic therapy. Boxes denote candidate populations; arrows denote progression and trial-design feasibility. Downward arrows (↓) indicate decreased activity, expression, or levels. Suggested endpoints include liver stiffness, fibrosis indices, inflammatory biomarkers, and incident HCC. Abbreviations: HCC, hepatocellular carcinoma; MASH, metabolic dysfunction-associated steatohepatitis.

Clinical translation of dietary polyphenols and nutraceutical strategies in hepatocellular carcinoma (HCC) depends on aligning three factors: (i) disease stage (primary prevention, secondary prevention after curative therapy, or adjunctive use in advanced disease), (ii) the dominant biological target (metabolic dysfunction, fibrosis, immune modulation, or gut–liver axis signaling), and (iii) the feasibility of measuring clinically meaningful endpoints within realistic trial designs [92]. Most nutritional interventions act as host-directed modifiers, influencing the disease substrate or treatment tolerance rather than functioning as direct anticancer agents. Consequently, trial design must distinguish between prevention-oriented and adjunctive objectives. Hard endpoints such as incident HCC in cirrhosis require large cohorts and extended follow-up. In contrast, intermediate markers, including liver stiffness, fibrosis indices, and inflammatory biomarkers, are more feasible but cannot be assumed to translate into cancer risk reduction without validation [92]. A stage-adapted framework for integrating nutraceuticals across prevention and treatment settings is summarized in Figure 7.

### 5.1. Positioning Nutraceuticals Within Contemporary HCC Therapeutic Pathways

Systemic therapy for unresectable HCC has shifted toward immune-based combinations. IMbrave150 established atezolizumab plus bevacizumab as first-line therapy, while HIMALAYA introduced dual checkpoint blockade as an alternative [93,94]. In the post-curative setting, IMbrave050 demonstrated improved recurrence-free survival with adjuvant atezolizumab plus bevacizumab in high-risk patients [95]. Within this framework, nutraceuticals are most plausibly positioned in three settings: (i) primary prevention or risk reduction in MASLD/MASH, viral hepatitis, and cirrhosis; (ii) secondary prevention following curative therapy, potentially as adjuncts to adjuvant systemic treatment; and (iii) adjunctive use in advanced HCC to modulate metabolic, fibrotic, or immune pathways and improve treatment tolerance, while accounting for drug–nutrient interactions [92,93,94,95]. Translation across all three settings is constrained by four interlocking limitations. (i) Bioavailability: native curcumin shows oral bioavailability of approximately 1% with rapid glucuronidation/sulfation, and resveratrol undergoes near-complete first-pass metabolism, yielding plasma concentrations far below those used in preclinical studies. EGCG and quercetin similarly show low and variable absorption. (ii) Standardization and product heterogeneity: commercial polyphenol preparations vary substantially in active compound content, excipients, and contaminants; pharmacopeial standards are absent for most agents, and label claims frequently diverge from analyzed content. (iii) Safety: dose-dependent hepatotoxicity has been documented for high-dose green tea catechin extracts (EFSA threshold ≥ 800 mg EGCG/day) and for turmeric/curcumin preparations (DILIN case series); high-dose vitamin E (≥400 IU/day) is associated with hemorrhagic stroke risk and a prostate cancer signal in SELECT. (iv) Drug–nutrient interactions: resveratrol and quercetin modulate CYP3A4, UGT, and efflux transporters (P-gp, BCRP) relevant to the metabolism of sorafenib, lenvatinib, and regorafenib; EGCG inhibits OATP1B-mediated uptake of several systemic agents; and immunomodulatory polyphenols may theoretically antagonize or enhance immune checkpoint inhibitors (atezolizumab, durvalumab, tremelimumab), with directionality unresolved [96,97,98,99,100]. These four constraints, rather than mechanistic plausibility, are the principal barriers to clinical translation [96,97].

### 5.2. Human Interventional Evidence

Human interventional data for polyphenols and nutraceuticals remain limited and focus primarily on chronic liver disease rather than HCC-specific outcomes. Legacy terminology (NAFLD, NASH) is retained below where the original trials used these designations, consistent with the convention. Curcumin trials in NAFLD/MASLD using bioavailability-enhanced formulations show short-term improvements in biochemical or imaging markers but provide no data on fibrosis durability or HCC incidence [101,102]. In biopsy-proven NASH, a randomized trial of high-dose silymarin failed to meet its primary endpoint but showed signals of reduced fibrosis, which are mechanistically relevant given the fibrosis–HCC risk relationship [103]. Clinical trial data for probiotics remain sparse; a perioperative probiotic trial before liver resection showed no clinical benefit, underscoring the need for rational strain selection, sufficient exposure, and mechanism-driven endpoints [104]. Relevant studies and ongoing trials are summarized in Table 3.

### 5.3. Combination Strategies and Adjunctive Use

Preclinical evidence suggests that polyphenols can sensitize tumor cells to cytotoxic, targeted, and immune-based therapies by modulating stress responses, apoptosis, ferroptosis, autophagy, and immune pathways. However, clinical data in HCC remain limited, and uncertainty persists regarding immune directionality and potential antagonism with immune checkpoint inhibitors [98,99,100]. Although low-toxicity adjuncts are appealing, modulation of cytochrome P450 enzymes, UGT pathways, and drug transporters raises concerns about unpredictable drug interactions, underscoring the need for mechanistically informed combination trials [96,97].

### 5.4. Safety, Dosing, and Regulatory Considerations

Nutraceuticals are often administered at pharmacologic doses in patients with chronic liver disease, raising safety concerns that are amplified by reduced hepatic reserve in cirrhosis. Herb- and supplement-induced liver injury (HILI/DILI) is increasingly recognized: turmeric/curcumin has been implicated in ten cases reported through the U.S. Drug-Induced Liver Injury Network (DILIN), several with HLA-B*35:01 association; high-dose green tea extracts enriched in EGCG (>800 mg/day) carry an EFSA-confirmed hepatotoxicity signal; and high-dose vitamin E (≥400 IU/day) carries cardiovascular and oncologic safety considerations. These findings argue for: (a) explicit dose justification with reference to no-observed-adverse-effect levels, (b) baseline and on-treatment ALT/AST/bilirubin monitoring, (c) exclusion of decompensated cirrhosis (Child–Pugh B/C) from early-phase trials, and (d) screening for concomitant hepatotoxic medications and supplements. Regulatory challenges further complicate evaluation: most jurisdictions classify polyphenol products as dietary supplements rather than drugs, thereby exempting them from pharmacopeial identity, purity, and potency testing. Product heterogeneity, adulteration, and inaccurate labeling are well documented. High-quality clinical studies should therefore treat nutraceuticals as investigational medicinal products with standardized formulations, certificates of analysis, batch traceability, contaminant testing (heavy metals, pesticides, and microbial), and stability data [96,97].

### 5.5. Trial Design Priorities and Future Directions

Future studies should focus on risk-stratified populations and mechanism-informed endpoints, including primary prevention in advanced fibrosis or cirrhosis, secondary prevention following curative therapy, and adjunctive trials in advanced HCC incorporating pharmacokinetic and immune profiling [92,95,99]. Rational combinations targeting fibrosis, bile acid signaling, and immune tone are likely to outperform single-agent approaches [98]. Ongoing registered trials will be critical for defining safety, biomarkers, and feasibility for larger studies [107,108].

### 5.6. Drug–Nutrient Interactions in Contemporary HCC Therapy

Given the IMbrave150, HIMALAYA, and IMbrave050 regimens now anchoring HCC management [93,94,95], polyphenol–drug interactions warrant explicit consideration. Multi-kinase inhibitors (sorafenib, lenvatinib, regorafenib) are CYP3A4 and UGT1A9 substrates and P-gp/BCRP transporter substrates; resveratrol, quercetin, and EGCG all modulate one or more of these pathways, raising the potential for altered systemic exposure. For monoclonal antibody-based therapies (atezolizumab, bevacizumab, durvalumab, tremelimumab), pharmacokinetic interactions are unlikely, but immunologic interactions remain unresolved: polyphenols with NF-κB/STAT3-suppressive activity could attenuate the inflammatory milieu required for ICI efficacy, while gut microbiome–modulating compounds may either enhance or impair ICI response depending on baseline dysbiosis [98,99]. Until prospective interaction studies are completed, concomitant high-dose nutraceutical use during systemic HCC therapy should be discouraged outside clinical trials An integrated summary of dietary polyphenols and non-polyphenol nutraceuticals, including their mechanistic targets, preclinical and clinical evidence, and major translational limitations in HCC, is provided in Table 4.

## 6. Conclusions

Hepatocellular carcinoma (HCC) arises predominantly from chronic liver injury, fibrosis, cirrhosis, and metabolic dysfunction, which also limit therapeutic reserve. Despite advances in systemic and immune-based therapies, recurrence, resistance, and liver-related morbidity remain barriers, highlighting the need for prevention-oriented strategies. The most substantial evidence supports dietary patterns rather than isolated supplements: plant-forward diets and coffee are associated with reduced HCC risk, whereas ultra-processed diets are associated with metabolic liver disease. Nutraceuticals remain mechanistically compelling but largely preclinical, supporting their use as preventive or adjunctive rather than standalone therapies. Rigorous, biomarker-guided trials with standardized formulations and safety oversight are essential for translation.

## Figures and Tables

**Table 1:** Non-polyphenol nutraceuticals with relevance to HCC prevention and adjunctive care.

Nutraceutical	Principal Mechanistic Targets Relevant to HCC	Most Consistent Human Signals	Potential HCC-Specific Applications	Key Cautions
Omega-3 PUFAs (EPA/DHA)	Reduced hepatic steatosis/lipotoxicity; anti-inflammatory lipid mediator shift; modulation of COX-2/β-catenin signaling in models [55,56,57]	Reduced liver fat and improved lipid parameters in NAFLD RCT meta-analyses [56,58,59,60,61]; observational inverse association with liver cancer risk in UK Biobank [58,59]	Prevention-oriented adjunct in MASLD/NASH; perioperative immunonutrition around hepatectomy [60,61]	Bleeding risk generally not increased overall, but high-dose purified EPA may modestly increase bleeding risk; individualize in cirrhosis/procedural contexts [62]
Vitamin E (α-tocopherol)	Antioxidant/lipid peroxidation reduction; attenuation of oxidative injury in NASH [63,64]	Histologic improvement in selected nondiabetic NASH (PIVENS) [63]; improved transplant-free survival/decompensation outcomes in the advanced fibrosis cohort [64]	Disease-substrate modification (NASH) to reduce progression toward cirrhosis-associated HCC	Safety concerns with high-dose long-term use (prostate cancer signal in SELECT †; hemorrhagic stroke risk signal); avoid indiscriminate use, select patients carefully [65,66]
Probiotics (strain-/mixture-specific)	Reduced endotoxemia; microbiome remodeling; immune modulation (e.g., Th17/IL-17) [67,68]	RCT meta-analysis supports benefits in cirrhosis outcomes and dysbiosis [69]; observational data suggest reduced HCC incidence in HBV-cirrhosis on antivirals [70]	Adjunct to improve cirrhosis-related inflammation/liver reserve; exploratory combination with systemic therapy (e.g., regorafenib tolerance/resistance in models) [71]	Product/strain variability; limited HCC endpoint trials; caution in severely immunocompromised or profoundly decompensated patients [69]

† SELECT used 400 IU/day vitamin E; PIVENS used 800 IU/day. Risk estimates from SELECT are not directly transferable to the PIVENS dosing regimen.

**Table 2:** Diet-responsive mechanistic axes in hepatocarcinogenesis and potential translational entry points.

Mechanistic Axis	Representative Nodes	Associated Dietary/Metabolic Exposures	Diet/Nutraceutical Modulators (Examples)	Translational Implication
Oxidative stress and redox buffering	ROS generation; Keap1-Nrf2 antioxidant program; glutathione pathways	Steatosis/lipotoxicity; chronic inflammation; alcohol; high-sugar diets [73,74]	Polyphenols that modulate Nrf2 and inflammatory signaling; antioxidant nutrients (context-dependent) [75,91]	Stage-specific strategy needed: prevention vs. established HCC; potential interaction with ferroptosis-based therapies [52,73]
Ferroptosis susceptibility	Lipid peroxidation; Nrf2-mediated cytoprotection; p62-Keap1 signaling	Lipid-rich tumor environment; oxidative pressure [52,73]	Compounds that alter lipid peroxidation balance (hypothesis-driven; requires biomarker guidance) [52,73]	Nrf2 activation may protect against ferroptosis; combining diet-derived agents with therapy requires mechanistic alignment [52,75]
Inflammatory oncogenic signaling	IL-6/JAK/STAT3; NF-κB; cytokine loops	NASH/MASH; obesity; chronic viral injury [74,78,79]	Polyphenols with anti-inflammatory actions; omega-3 and pro-resolving lipid mediators (immunometabolic effects) [78,82]	Candidate axis for combined host-directed + tumor-directed strategies; supports etiology-based stratification [81,82]
Gut-liver axis	Dysbiosis; barrier dysfunction; bile acid signaling; microbial metabolites	Low fiber; ultra-processed diets; high sugar; cirrhosis-related dysbiosis [83,85]	Fiber/prebiotics; probiotics; polyphenols influencing microbiome; bile acid-directed strategies [84,90]	Microbiome features associate with ICI response; diet-based microbiome modulation may become an adjunctive lever [85]
Lipid metabolism and de novo lipogenesis (DNL)	SREBP-1; ACC; FASN; SCD; *AKT-mTOR*-lipogenesis signaling	Fructose excess; caloric excess; saturated fat; insulin resistance [87,90]	Mediterranean-style dietary patterns; omega-3; lipogenesis inhibitors (drug class); reduced added sugars [89,90]	Human data show de novo lipogenesis (DNL) is druggable; metabolic targeting could complement prevention and potentially therapy [87,88]

**Table 3:** Overview of selected human interventional studies evaluating polyphenol-based and non-polyphenol nutraceuticals for HCC prevention.

Intervention	Study Type/Population	Dose & Duration	Key Outcomes	Translational Notes
Phytosomal curcumin	RCT in NAFLD	Formulation-specific; short-term	Reported improvement in NAFLD-related measures vs. placebo	Surrogate outcomes only; does not address fibrosis durability or HCC endpoints [101]
Curcuminoids + piperine	RCT in ultrasound-defined NAFLD	500 mg curcuminoids + 5 mg piperine daily; 12 weeks	Improved NAFLD severity on sonography; changes in liver enzymes vs. placebo	Illustrates formulation/bioavailability importance; piperine may influence drug metabolism and safety [102,105]
Silymarin (high-dose)	Double-blind RCT in biopsy-proven NASH	700 mg three times daily; 48 weeks	Primary NAS endpoint not met; fibrosis reduction signal and improved liver stiffness/fibrosis indices.	Fibrosis-focused outcomes are most relevant to long-term HCC risk biology [103]
Probiotics pre-hepatectomy	RCT in chronic liver disease with resectable HCC	14 days preop	No endotoxin or clinical outcome benefit vs. placebo; cytokine changes	Negative/neutral trial highlights need for strain selection, duration optimization, and mechanistic endpoints [104]
EGCG for HCC prevention in cirrhosis	Preclinical and translational evidence	Protocol-defined	HCC prevention intent	A preclinical and translational review; no confirmed registered clinical trial identified at time of writing. Trial registration should be verified before submission [106,107].
Probiotics for HCC prevention in cirrhosis	Ongoing/registered trial	Protocol-defined	HCC prevention intent	A review of microbiota modulation and immunotherapy; no confirmed registered clinical trial identified at time of writing. Trial registration should be verified before submission [108].

**Table 4:** Integrated summary of dietary polyphenols and non-polyphenol nutraceuticals evaluated in this review. Classification, principal mechanisms in hepatocarcinogenesis, evidence tier, and major translational limitations.

Compound	Class	Principal Mechanisms in HCC	In Vitro Evidence	In Vivo (Animal) Evidence	Human/Clinical Evidence	Major Limitations
**Curcumin**	Curcuminoid (non-flavonoid)	↓ NF-κB, STAT3, PI3K/AKT, TGF-β/SMAD, Wnt/β-catenin; G2/M arrest; mitochondrial apoptosis [21,22]	Yes (HepG2, Hep3B, Huh7) [18,21]	Yes (DEN, xenograft rodent models) [21,23]	NAFLD/MASLD RCTs (surrogate endpoints) only; no HCC trials [102,103]	~1% oral bioavailability; turmeric DILI cases (DILIN, HLA-B*35:01); CYP3A4/UGT modulation
**Resveratrol**	Stilbene (non-flavonoid)	↑ p53, PTEN; ↓ PI3K/AKT, STAT3, NF-κB; apoptosis + autophagy [26,27,28]	Yes [26,27,28]	Yes (rodent HCC models) [25]	No HCC trials; limited NAFLD data	First-pass metabolism; CYP3A4 inhibition (sorafenib/lenvatinib risk) [100,101]
**EGCG**	Flavan-3-ol (flavonoid)	↓ RTK, PI3K/AKT, ERK; mitochondrial apoptosis; HSC senescence [20,30,31]	Yes	Yes (DEN; sorafenib synergy) [31,32]	No HCC trials	EFSA hepatotoxicity threshold ≥800 mg/day; OATP1B inhibition
**Quercetin**	Flavonol (flavonoid)	↓ NF-κB, STAT3, PI3K/AKT, JAK2/STAT3; ↑ ROS, caspases [33,34]	Yes (HepG2, LM3)	Yes (rodent) [34]	No HCC trials	Low bioavailability; CYP/P-gp modulation
**Luteolin**	Flavone (flavonoid)	↑ p53, Fas/FasL; ↓ TGF-β, PI3K/AKT, NF-κB, JAK/STAT, Wnt/β-catenin [35,36]	Yes (HepG2)	Limited rodent data	No HCC trials	Poor solubility; minimal human PK data
**Baicalein**	Flavone (flavonoid)	ER stress, caspase activation, LC3/Beclin-1 autophagy [37,38]	Yes	Limited	No HCC trials	Poor bioavailability; standardization
**Naringenin**	Flavanone (flavonoid)	ROS-dependent mitochondrial apoptosis; ↓ JAK2/STAT3 [40,41]	Yes (HepG2)	Limited (nanoparticle formulations) [42]	No HCC trials	Low absorption; nanoformulation PK unestablished in humans
**Silymarin/Silibinin**	Flavonolignan	Antiproliferative; ↑ p53/p21/p27; hepatoprotective; antioxidant [43,44,45]	Yes (HepG2)	Yes	NASH RCT (high-dose; primary endpoint missed, fibrosis signal) [104]; no HCC trials	Variable product composition; high-dose required
**Omega-3 PUFAs (EPA/DHA)**	Long-chain polyunsaturated fatty acids	↓ COX-2, Wnt/β-catenin; pro-resolving lipid mediators; reduced steatosis/lipotoxicity [56,66,109]	Yes	Yes	NAFLD RCT meta-analyses [55,67,68]; UK Biobank inverse association with liver cancer [57,69]; perioperative hepatectomy meta-analyses [58,110]	High-dose EPA bleeding risk [70]; supplement vs. dietary equivalence unclear
**Vitamin E (α-tocopherol)**	Lipid-soluble antioxidant	↓ lipid peroxidation; attenuates NASH oxidative injury [59,60]	—	—	PIVENS RCT (800 IU/day; histologic improvement in non-diabetic, non-cirrhotic NASH) [59]; observational survival benefit in advanced fibrosis [60]	Hemorrhagic stroke risk [61]; SELECT prostate cancer signal at 400 IU/day [71]; PIVENS excluded cirrhosis
**Probiotics (strain-specific)**	Live microorganisms	↓ endotoxemia/LPS; microbiome remodeling; bile acid signaling; Th17/IL-17 modulation [72,73]	—	Yes (Lactobacillus rhssamnosus rodent HCC model) [74]	RCT meta-analysis in cirrhosis (HE, dysbiosis) [65]; observational HBV-cirrhosis dose–response with HCC incidence [64]; pre-hepatectomy RCT neutral [105]	Strain/product variability; no HCC-endpoint RCTs; caution in decompensated/immunocompromised patients

**Footnotes:** Evidence tier reflects highest-quality study type available in HCC or its immediate precursor (MASLD/MASH/cirrhosis); “No HCC trials” indicates absence of registered, completed interventional studies with HCC-related endpoints at time of writing. ↑ indicates increased expression, activation, or levels; ↓ indicates decreased expression, activation, or levels. Abbreviations: DEN, diethylnitrosamine; DILI, drug-induced liver injury; HE, hepatic encephalopathy; HSC, hepatic stellate cell; PK, pharmacokinetics; RCT, randomized controlled trial.

## Data Availability

No new data were created or analyzed in this study. Data sharing is not applicable to this article.

## Abbreviations

AASLD	American Association for the Study of Liver Diseases
ACC	Acetyl-CoA Carboxylase
ACLY	ATP-Citrate Lyase
AKT	Protein Kinase B
BCRP	Breast Cancer Resistance Protein
Bcl-2	B-cell Lymphoma 2
CDK	Cyclin-Dependent Kinase
COX-2	Cyclooxygenase-2
CXCR2	C-X-C Motif Chemokine Receptor 2
CYP3A4	Cytochrome P450 3A4
DEN	Diethylnitrosamine
DHA	Docosahexaenoic Acid
DNL	De Novo Lipogenesis
eEF2K	Eukaryotic Elongation Factor 2 Kinase
EGCG	Epigallocatechin-3-Gallate
EPA	Eicosapentaenoic Acid
ER	Endoplasmic Reticulum
ERK	Extracellular Signal-Regulated Kinase
FASN	Fatty Acid Synthase
FXR	Farnesoid X Receptor
HBV	Hepatitis B Virus
HCC	Hepatocellular Carcinoma
HCV	Hepatitis C Virus
ICI	Immune Checkpoint Inhibitor
IL-6	Interleukin-6
IL-8	Interleukin-8
IL-17	Interleukin-17
JAK	Janus Kinase
Keap1	Kelch-like ECH-Associated Protein 1
LC3	Microtubule-Associated Protein 1 Light Chain 3
MAPK	Mitogen-Activated Protein Kinase
MASH	Metabolic Dysfunction-Associated Steatohepatitis
MASLD	Metabolic Dysfunction-Associated Steatotic Liver Disease
mTOR	Mechanistic Target of Rapamycin
NAFLD	Non-Alcoholic Fatty Liver Disease
NAS	NAFLD Activity Score
NASH	Non-Alcoholic Steatohepatitis
NF-κB	Nuclear Factor Kappa B
Nrf2	Nuclear Factor Erythroid 2-Related Factor 2
PD-1	Programmed Cell Death Protein 1
P-gp	P-glycoprotein
PI3K	Phosphoinositide 3-Kinase
PIVENS	Pioglitazone vs. Vitamin E vs. Placebo for NASH Trial
PTEN	Phosphatase and Tensin Homolog
PUFA	Polyunsaturated Fatty Acid
RCT	Randomized Controlled Trial
ROS	Reactive Oxygen Species
RSV	Resveratrol
SCD1	Stearoyl-CoA Desaturase 1
SELECT	Selenium and Vitamin E Cancer Prevention Trial
SMAD	Suppressor of Mothers Against Decapentaplegic
SREBP-1	Sterol Regulatory Element-Binding Protein 1
STAT3	Signal Transducer and Activator of Transcription 3
TGF-β	Transforming Growth Factor Beta
TGR5	Takeda G-Protein-Coupled Receptor 5
Th17	T Helper 17 Cell
UGT	UDP-Glucuronosyltransferase
Wnt	Wingless-related Integration Site
